# Gasotransmitters: novel regulators of ion channels and transporters

**DOI:** 10.3389/fphys.2013.00027

**Published:** 2013-02-21

**Authors:** Mike Althaus, Wolfgang G. Clauss

**Affiliations:** Department of Molecular Cell Physiology, Institute of Animal Physiology, Justus-Liebig University of GiessenGiessen, Germany

More than 25 years ago, it was a big surprise for physiologists that nitric oxide (NO) was identified as the *endothelium derived relaxing factor* which is responsible for endothelium-induced smooth muscle relaxation (Ignarro et al., 1987). Until then, small gaseous molecules were simply regarded as byproducts of cellular metabolism which were unlikely to be of any physiological relevance. The discovery that NO was synthesized by specific enzymes (NO-synthases), upon stimulation by specific, physiologically relevant stimuli (e.g., acetylcholine stimulation of endothelial cells), as well as the fact that it acted on specific cellular targets (e.g., soluble guanylate cyclase), set the course for numerous studies which investigated the physiological roles of gaseous signaling molecules—in other words, *gasotransmitters* (Wang, 2002).

Aside from NO, there are two more gases which subsequently have been identified as gasotransmitters: carbon monoxide (CO) (Wu and Wang, 2005) and hydrogen sulfide (H_2_S) (Abe and Kimura, 1996; Wang, 2002). Although the concept of a gas being involved in cellular signaling processes was already accepted since the discovery of NO, the new candidates CO and H_2_S were initially met with skepticism—especially since they were well-known for their high toxicity and regarded as environmental chemical threats. However, in concert with the principle of Paracelsus that *“everything is toxic- it just depends on the dose,”* it is nowadays accepted that small amounts of CO and H_2_S are of physiological relevance and are even endogenously produced in human cells (Wu and Wang, 2005; Wang, 2012).

All three gasotransmitters are produced in human cells by specific enzymes: NO is produced by NO-synthases (NOS), originating from the amino acid L-arginine. There are three NOS-isoforms, NOS1-3, which have been originally termed neuronal, inducible and endothelial NOS, respectively (Garvin et al., 2011). CO is generated by heme oxygenases (HO) within heme degradation (Wu and Wang, 2005). There are also three heme oxygenases (HO-1-3) (Yoshida et al., 1974; Maines et al., 1986; McCoubrey et al., 1997), including an inducible HO-1 and a constitutively active HO-2. H_2_S is mainly produced within the metabolism of L-cysteine by the enzymes cystathionine-β-synthase (CBS), cystathionine-γ-lyase (CSE or CTH) and 3-mercaptopyruvate sufurtransferase (3-MST) (Wang, 2012). Recently, Kimura's group described an additional pathway for endogenous H_2_S production, which involves D-cysteine, 3-MST, and D-amino acid oxidase (Shibuya et al., 2013).

Despite the knowledge of the substrates and specific enzymes which are involved in gasotransmitter production, the precise endogenous concentrations of NO, CO, and H_2_S have still not been sufficiently determined. The measurement of endogenous gas concentrations is limited by the accuracy and specificity of currently available methods (Olson, 2013), the reactivity and short half-life of some gases (NO) (Wall et al., 2012), and simply the fact that gases are volatile and rapidly disappear during the preparation procedures of biological samples. As highlighted within this research topic issue, it is crucial—and probably most challenging—to precisely quantify endogenous concentrations of NO, CO, and H_2_S (Kimura, 2012; Peers, 2012; Olson, 2013).

Although precise endogenous concentrations of gasotransmitters remain to be determined, numerous studies investigated the physiological effects of those gaseous signaling molecules in almost every organ system [as summarized in excellent review articles such as Olson (2011), Wu and Wang (2005), and Wang (2012)]. Common molecular targets for all gasotransmitters are ion channels and transporters. Changes in the activity of membrane-located ion channels/transporters are involved in the majority of physiological processes which are regulated by gasotransmitters. This allows the gases to specifically act at the interface of cell-environment interactions, electrolyte homeostasis and electrochemical communication—thus allowing the regulation of numerous physiological processes in cells and tissues.

This research topic summarizes currently available data on the regulation of ion channels and transporters by NO, CO, and H_2_S. Excellent review and research articles highlight the importance of gasotransmitter/ion channel interactions for vegetative physiology (Althaus, 2012; Peers, 2012; Pouokam and Diener, 2012), neurophysiology (Njie-Mbye et al., 2012; Peers, 2012; Takahashi et al., 2012; Wang et al., 2012), and explain gasotransmitter-induced signaling mechanisms (Wall et al., 2012). Furthermore, opinion articles from pioneers in gasotransmitter research (Kimura, 2012; Peers, 2012; Olson, 2013) give perspectives on important routes which should be followed in this field. With the articles included in this issue we wish to highlight the importance of ion channel/transporter regulation by gaseous signaling molecules and wish to stimulate future research in this exciting area of physiology.
